# Prognostic value of future liver remnant LU15 index of ^99m^Tc-galactosyl serum albumin scintigraphy for predicting posthepatectomy liver failure

**DOI:** 10.1371/journal.pone.0247675

**Published:** 2021-02-23

**Authors:** Koichi Tomita, Naokazu Chiba, Shigeto Ochiai, Takahiro Gunji, Kosuke Hikita, Toshimichi Kobayashi, Toru Sano, Yuta Abe, Kiyoshi Koizumi, Motohide Shimazu, Shigeyuki Kawachi

**Affiliations:** 1 Department of Digestive and Transplantation Surgery, Tokyo Medical University Hachioji Medical Center, Tokyo, Japan; 2 Department of Surgery, Keio University School of Medicine, Tokyo, Japan; 3 Department of Radiology, Tokyo Medical University Hachioji Medical Center, Tokyo, Japan; 4 Department of Surgery, Tama Kyuryo Hospital, Tokyo, Japan; Babasaheb Bhimrao Ambedkar University (A Central University), INDIA

## Abstract

There is no gold standard indicator that is currently used to predict posthepatectomy liver failure (PHLF). A novel indicator of liver function, the LU15 index of ^99m^Tc-galactosyl serum albumin (GSA) scintigraphy, refers to the liver uptake ratio over a 15-min interval. We aimed to evaluate the usefulness of the future liver remnant (FLR)-LU15 in predicting PHLF. The clinical data of 102 patients (70 males and 32 females; median age, 70 years) who underwent liver resection between January 2011 and August 2019 were analyzed. The FLR-LU15 was calculated by a fusion of simulated 3-dimensional images and ^99m^Tc-GSA scintigraphy. PHLF was determined according to the definition of the International Study Group of Liver Surgery. The FLR-LU15 was an independent risk factor for PHLF ≥ Grade B according to multivariate analysis, and its value correlated with the PHLF grade. The area under the receiver operating characteristic curve of the FLR-LU15 for PHLF ≥ Grade B was 0.816 (95% confidence interval, 0.704–0.929), which was better than that of other indicators. When the cut-off value of FLR-LU15 was set at 16.7, the sensitivity was 86.7%, specificity was 74.7%, and odds ratio was 19.2 (95% confidence interval, 4.0–90.9), all of which were superior to other indicators. If the cut-off value was 13, the positive predictive value was 57.1%. The FLR-LU15 is a useful predictor of PHLF and may be more reliable than other predictors.

## Introduction

Liver resection is the mainstay of treatment for hepatocellular carcinoma (HCC), liver metastases of colon cancer, and other liver diseases in selected patients. As a result of improvements in perioperative patient care and surgical techniques, the operative outcomes of liver resection have improved substantially in recent years. Nevertheless, postoperative morbidity and mortality still exist, with posthepatectomy liver failure (PHLF) being one of the most crucial postoperative complications [1].

PHLF occurs in up to 9% of patients undergoing hepatectomy [2, 3]. A national clinical database in Japan reported that the incidence of PHLF ranged from 0 to 11.8% depending on the surgical procedure [4]. In patients with liver cirrhosis who underwent hepatectomy for HCC, mortality due to PHLF was reported to be 2.9–3.3%, [5] though the frequency of PHLF was also reported to be 1–16% in noncirrhotic patients who underwent hepatectomy for colorectal liver metastasis [6]. The precise assessment of liver function strongly assists in the prediction and prevention of PHLF.

Although a number of values, indices, and criteria have been reported for the evaluation of liver function and the prediction of PHLF, there is no recognized gold standard. Furthermore, most indices can only calculate total liver function and cannot estimate future liver remnant (FLR) function precisely. Recent studies have reported the utility of ^99m^Tc-galactosyl human serum albumin (GSA) scintigraphy in predicting PHLF [7, 8]. One of the greatest benefits of ^99m^Tc-GSA scintigraphy is that it can calculate the function of specific parts of the liver [9, 10]. ^99m^Tc-GSA scintigraphy can be performed not only for the total liver but also for the FLR by quantifying the accumulation of ^99m^Tc-GSA in the residual liver. In addition, ^99m^Tc-GSA scintigraphy can be performed in both normal liver and cirrhosis in the same way.

Indices of ^99^mTc-GSA scintigraphy include the LHL15, defined as the ratio of uptake by the liver to that by the liver and heart combined at 15 min (L15/(H15 + L15)); HH15, defined as the ratio of uptake by the heart at 15 min to that at 3 min (H15/H3) [11]; LU15, defined as the ratio of uptake by the liver at 15 min to the injected dose of ^99m^Tc-GSA [12]; GSA index, defined as LHL15/HH15. Of these indices, HH15 and LHL15 are commonly used [13]. Compared to the other indices, the usefulness of the LU-15, including the FLR-LU15 to predict PHLF has not been well studied.

Therefore, in this study, we measured the clinical usefulness of the FLR-LU15 index of ^99m^Tc-GSA scintigraphy to predict PHLF in patients undergoing liver resection. The optimum cut-off value for predicting PHLF was also calculated.

## Methods

The procedures were approved by the institutional review board of Tokyo Medical University Hachioji Medical Center (H-175) and in accordance with the Helsinki Declaration of 1975, as revised in 1983. Written informed consent was obtained from all patients.

### Subjects

**Fig 1:**
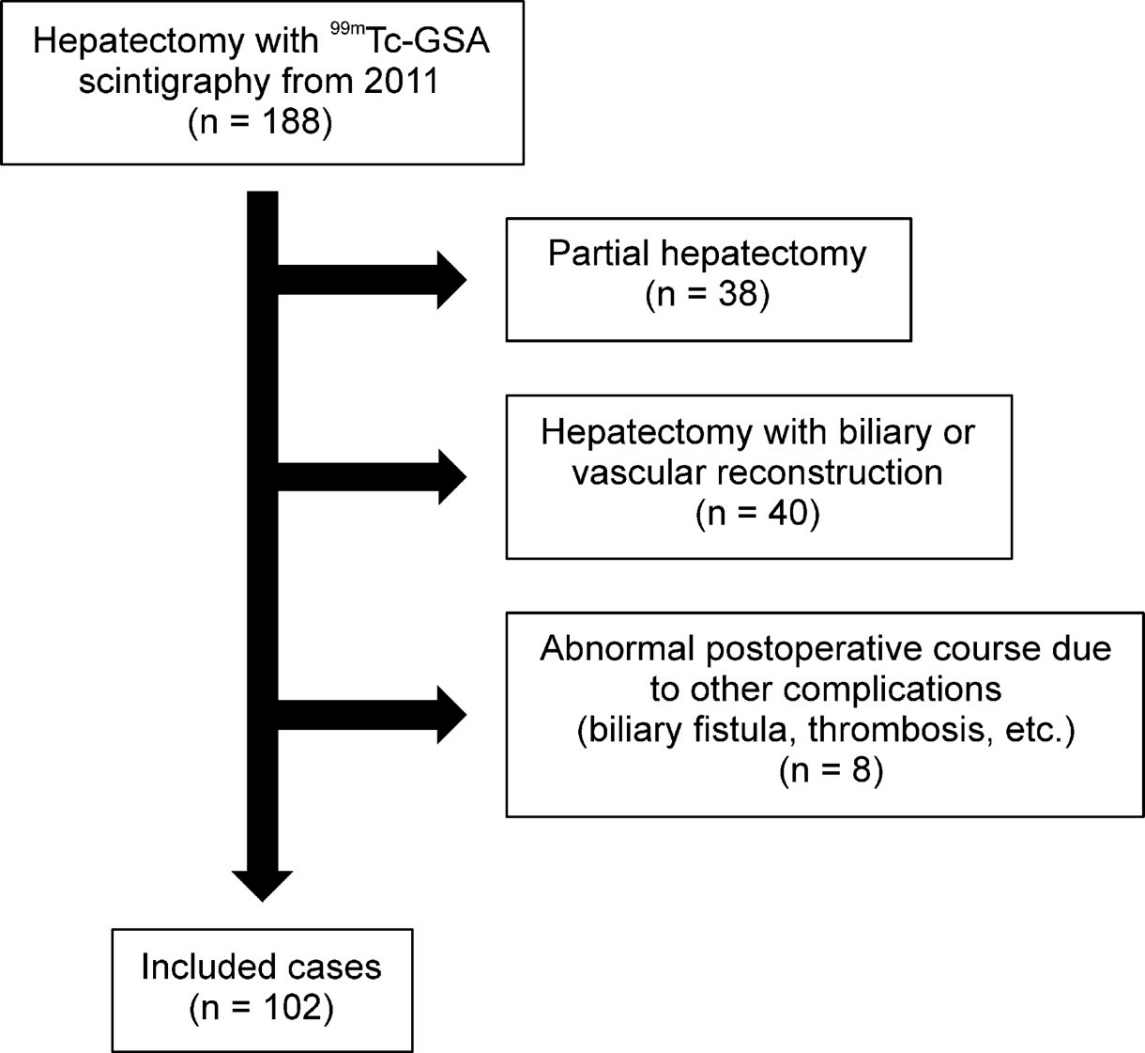
Study flowchart. Study patient inclusion/exclusion flowchart.

We retrospectively analyzed the clinical data of 188 patients who underwent liver resection at the Tokyo Medical University Hachioji Medical Center between January 2011 and August 2019. The patients underwent ^99m^Tc-GSA scintigraphy before surgery. These patients were part of the study population of our previous retrospective study that evaluated the usefulness of the LU15 index in ^99m^Tc-GSA scintigraphy for predicting PHLF [14, 15]. We excluded patients who underwent partial hepatectomy (n = 38), because it rarely occurs in PHLF. We also excluded hepatectomy with biliary or vascular reconstruction (n = 40) and those for whom evaluation of postoperative liver failure was difficult because of complications other than liver failure, such as biliary fistula or portal vein thrombosis (n = 8). After excluding these cases, the remaining 102 cases were included in this study (Fig 1).

The clinical data analyzed in the present study included age, sex, tumor diagnosis, blood examination (total bilirubin, albumin, international normalized ratio of prothrombin time [PT-INR], aspartate transaminase, and alanine transaminase), liver function tests (Child-Pugh classification), indocyanine green retention rate at 15 min [ICG-R15], Makuuchi criteria, and albumin-bilirubin (ALBI) score, procedure of hepatectomy, history of percutaneous transhepatic portal vein embolization (PTPE), computed tomography including three-dimensional (3D) images for preoperative assessment, the indices of ^99m^Tc-GSA scintigraphy (HH15, LHL15, LU15, and GSA), operative time, blood loss, postoperative complications including PHLF, and postoperative stay. The ICG clearance rate (KICG) was not measured at our institution.

### ^99m^Tc-GSA scintigraphy

**Fig 2:**
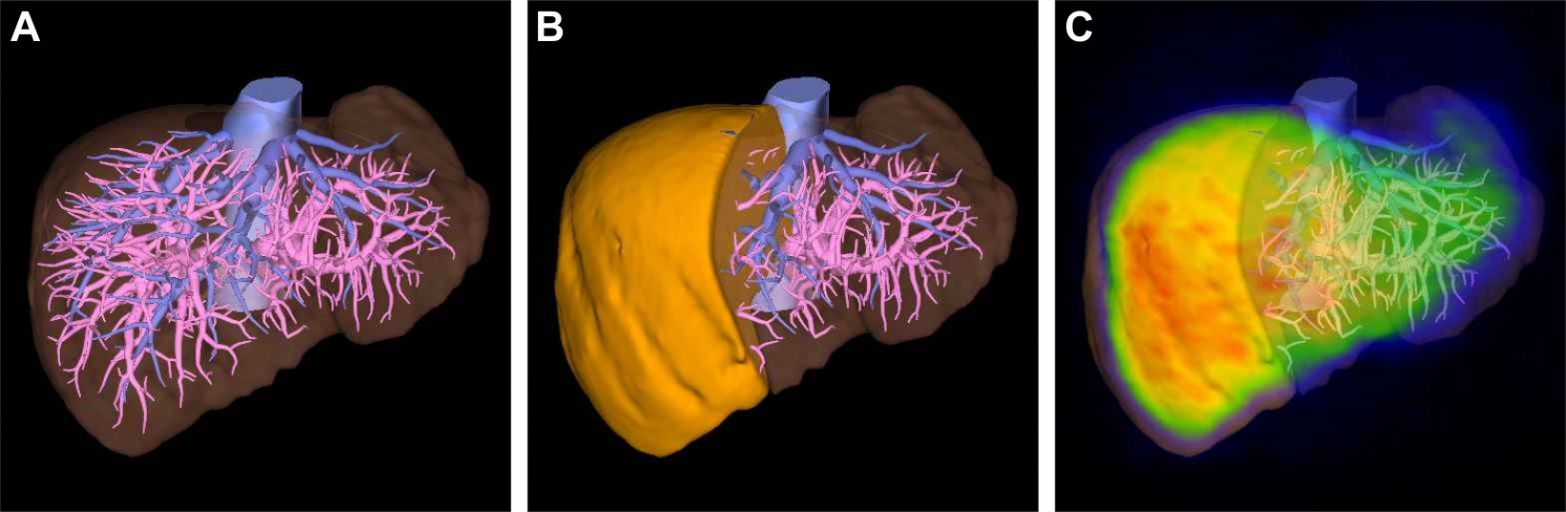
A sample case of the future liver remnant (FLR) ^99m^Tc-galactosyl serum albumin (GSA) scintigraphy calculation. The three-dimensional image is simulated by a volume analyzer system (a), and the total liver volume is 1283 mL. In the case of a right hepatectomy, the FLR volume is 533 mL (41.5%) (b). The simulated image is fused with ^99m^Tc-GSA scintigraphy, and the FLR count of ^99m^Tc-GSA is 34.8% (c). The FLR of each index of ^99m^Tc-GSA scintigraphy can be calculated by multiplying the total liver value by 34.8%.

^99m^Tc-GSA scintigraphy was performed for all subjects before surgery using PRISM-IRIX (Picker Corp., Cleveland, Ohio, USA/Shimadzu Corp., Kyoto, Japan) until October 2012 and then BrightView (Philips Medical Systems, Cleveland, OH) thereafter. These were carefully calibrated to obtain the same result when the systems were changed over. All patients received 185 MBq of ^99^mTc-GSA (3 mg of GSA; Nihon Medi-Physics, Nishinomiya, Japan). After administration, single-photon emission computed tomography (SPECT) and dynamic imaging were performed. The scintigraphy requires approximately 40–50 min in total for each patient. There were no apparent contraindications when performing this test. Liver function in ^99m^Tc-GSA scintigraphy included the HH15, LHL15, LU15, and the GSA indices. Of these indices, the LHL15, LU15, and GSA gave higher values with improved liver function, whereas the HH15 showed lower values with improved liver function. Both total liver and FLR of these indices were obtained, except for the HH15. The HH15 can be calculated only for the total liver. The FLR count of ^99m^Tc-GSA scintigraphy was calculated by fusing the 3D computed tomography images and ^99m^Tc-GSA scintigraphy using a volume analyzer system (Synapse Vincent, Fujifilm, Japan). A case of fusion is shown in Fig 2.

### Definition of PHLF

PHLF was diagnosed based on the International Study Group of Liver Surgery definition [16]. Elevated PT-INR with concomitant hyperbilirubinemia on or after postoperative day 5 was diagnosed as PHLF. The severity of the PHLF was graded as follows: Grade 0, no PHLF; Grade A, PHLF that required no change in the patient’s clinical management; Grade B, PHLF that required a deviation from the regular course but did not require invasive therapy; and Grade C, PHLF that required invasive treatment.

### Statistical analyses

All statistical analyses were performed using SPSS software (version 25.0, SPSS Inc., Chicago, IL, USA). Continuous variables were expressed as mean ± standard deviation or median with range, and the Mann-Whitney U-test was used to compare groups. Categorical variables were compared using the Chi-square test or Fisher’s exact test, as required. The differences in FLR-LU15 among PHLF grades and between each grade were compared using the Kruskal–Wallis test and the Mann–Whitney U test, respectively.

In the multivariate analysis, the variables were selected based on the univariate analysis, and logistic regression analysis was performed with the forward selection (likelihood ratio). The predictive values of PHLF ≥ Grade B were measured using receiver operating characteristic (ROC) analysis, and the area under the ROC curve (AUROC) was calculated. Additionally, the cut-off values of LHL15, LU15, and GSA indices were obtained from ROC analysis. Regarding LU15, the cut-off value was also evaluated in terms of positive predictive value (PPV), negative predictive value (NPV), and the Youden index: sensitivity—(1-specificity). Both these indices and the Makuuchi criteria were compared using the Chi-square test or Fisher’s exact test, as required. Statistical tests were two-tailed, and significance was set at *P*-values < 0.05.

We calculated that a sample size of 100 patients would give a power of 80% to compare the value of FLR-LU15 between PHLF < Grade B and ≥ Grade B when using *t*-tests. This sample size considered an expected incidence of PHLF of 15%, an effect size of 0.8, and a two-sided alpha risk of 0.05.

## Results

### Patient characteristics

Patient characteristics and comparisons between PHLF < Grade B and ≥ Grade B are summarized in Table 1. There were more male and HCC patients in the PHLF ≥ Grade B group (*P* = 0.02 and *P* < 0.01, respectively). There were no patients with a Child-Pugh Grade C classification.

**Table 1:** Characteristics of the study population.

			All cases	PHLF < Grade B	PHLF ≥ Grade B	
			N = 102	N = 87	N = 15	*P-*value
General background						
	Age	y	70 (21–85)	69 (26–82)	72 (21–85)	0.23
	Sex	Male	70 (68.6%)	56 (64.4%)	14 (93.3%)	0.02
		Female	32 (31.4%)	31 (35.6%)	1 (6.7%)	
	Diagnosis	HCC	58 (56.9%)	44 (50.6%)	14 (93.3%)	< 0.01
		Liver metastasis	26 (25.5%)	26 (29.9%)	0 (0.0%)	
		Others	18 (17.6%)	17 (19.5%)	1 (6.7%)	–
	Preoperative PTPE		13 (13.4%)	12 (14.3%)	1 (7.7%)	0.45
Preoperative blood test						
	Total bilirubin	mg/dL	0.68 ± 0.29	0.65 ± 0.29	0.81 ± 0.30	0.07
	Albumin	g/dL	3.7 ± 0.4	3.7 ± 0.4	3.7 ± 0.6	0.79
	Prothrombin	%	103 ± 13	104 ± 12	95 ± 12	0.06
	Aspartate transaminase	IU/L	38 ± 22	35 ± 21	50 ± 25	0.05
	Alanine transaminase	IU/L	33 ± 28	29 ± 22	53 ± 43	< 0.01
Liver function test						
	Child–Pugh classification	A	100 (98.0%)	89 (98.9%)	14 (93.3%)	0.27
		B	2 (2.0%)	1 (1.1%)	1 (6.7%)	
	Indocyanine green	R15	11.2 ± 6.6	10.7 ± 6.4	14.1 ± 7.2	0.11
	Makuuchi criteria	Within	79 (77.5%)	70 (80.5%)	9 (60.0%)	0.08
		Without	23 (22.5%)	17 (19.5%)	6 (40.0%)	
	ALBI score		–2.48 ± 0.32	–2.49 ± 0.28	–2.39 ± 0.48	0.41
	^99m^Tc–GSA scintigraphy	LHL15	0.91 ± 0.04	0.91 ± 0.04	0.90 ± 0.05	0.67
		HH15	0.63 ± 0.10	0.62 ± 0.10	0.67 ± 0.08	0.08
		LU15	27.2 ± 6.1	27.6 ± 6.2	24.8 ± 5.1	0.07
		GSA index	1.48 ± 0.28	1.50 ± 0.28	1.37 ± 0.21	0.05
Future liver remnant function						
	^99m^Tc–GSA scintigraphy	Remnant count (%)	69.7 ± 14.2	71.0 ± 14.2	62.3 ± 12.6	0.03
		LHL15	0.63 ± 0.13	0.64 ± 0.13	0.56 ± 0.12	0.02
		LU15	18.7 ± 4.9	19.3 ± 4.9	15.0 ± 2.7	< 0.01
		GSA index	1.03 ± 0.31	1.07 ± 0.31	0.85 ± 0.20	< 0.01
Surgical factor						
	Procedure	Segmentectomy	20 (19.6%)	14 (16.1%)	6 (40.0%)	0.10
		Sectionectomy	26 (25.5%)	25 (28.7%)	1 (6.7%)	
		Hemihepatectomy	51 (50.0%)	44 (50.6%)	7 (46.7%)	
		Trisectionectomy	5 (4.9%)	4 (4.6%)	1 (6.7%)	
	Operative time	min	317 (70–780)	313 (70–575)	492 (246–780)	< 0.01
	Blood loss	g	350 (0–3840)	297 (0–2110)	505 (60–3840)	0.02
Surgical outcome						
	PHLF Grade	0: A: B: C	60: 27: 12: 3	60: 27: 0: 0	0: 0: 12: 3	–
	Postoperative stay	Days	14 (8–116)	14 (8–67)	22 (13–116)	< 0.01

Continuous variables are expressed as mean values ± standard deviations or medians with ranges. Categorical variables are expressed as number of patients.

ALBI: Albumin–bilirubin; GSA index: LHL15/HH15; HCC: Hepatocellular carcinoma; HH15: Ratio of uptake by the heart at 15 min to that at 3 min; LHL15: Ratio of uptake by the liver to that by the liver and heart combined at 15 min; LU15: Ratio of uptake by the liver at 15 min to the injected dose of ^99^mTc-GSA; PHLF: Posthepatectomy liver failure; PTPE: Percutaneous transhepatic portal vein embolization.

Following ^99m^Tc-GSA scintigraphy, the mean value of the LHL15, LU15, and GSA indices of the total liver were 0.91, 27.2, and 1.48, respectively. After surgical simulation by the volume analyzer system, these indices decreased or increased to 0.63, 18.7, and 1.03, respectively. No liver function tests of the total liver showed significant differences between PHLF < Grade B and ≥ Grade B groups, whereas the LHL15, LU15, and GSA indices of ^99m^Tc-GSA scintigraphy showed significantly lower values in the PHLF ≥ Grade B group (*P* = 0.02, *P* < 0.01, and *P* = 0.02, respectively).

There were no significant differences in the procedure of hepatectomy between the PHLF < Grade B and ≥ Grade B groups. The median operative time and median blood loss were significantly higher in the PHLF ≥ Grade B group (*P* = 0.03 and *P* = 0.02, respectively).

In the postoperative course, the PHLF was Grade 0 in 60 patients, Grade A in 27 patients, Grade B in 12 patients, and Grade C in three patients. The median postoperative stay was 14 days (range 8–116 days) and was significantly longer in the PHLF ≥ Grade B group (*P* < 0.01).

### Multivariate analysis for PHLF ≥ Grade B

Multivariate regression analysis was performed between variables, with a statistically significant difference following univariate analysis (Table 1). The FLR count (odds ratio [OR] 1.104, 95% confidence interval [CI] 1.025–1.189, *P* = 0.01) and the FLR-LU15 (OR 1.495, 95% CI 1.105–2.020, *P* = 0.01) were independent risk factors for PHLF ≥ Grade B (Table 2).

**Table 2:** Results of multivariate analysis for PHLF ≥ Grade B.

		*P-*value	Odds Ratio	95% CI
General background				
	Sex	0.23		
	Diagnosis	0.35		
Preoperative blood examination				
	Aspartate transaminase	0.21		
	Alanine transaminase	0.13		
Future liver remnant values of ^99m^Tc-GSA scintigraphy				
	Remnant count	0.01	1.104	1.025–1.189
	LHL15	0.17		
	LU15	0.01	1.495	1.105–2.020
	GSA index	0.93		

CI: Confidence interval; GSA index: LHL15/HH15; LHL15: Ratio of uptake by the liver to that by the liver and heart combined at 15 min; LU15: Ratio of uptake by the liver at 15 min to the injected dose of ^99^mTc-GSA; PHLF: Posthepatectomy liver failure.

### Relationship between FLR-LU15 index and PHLF

**Fig 3:**
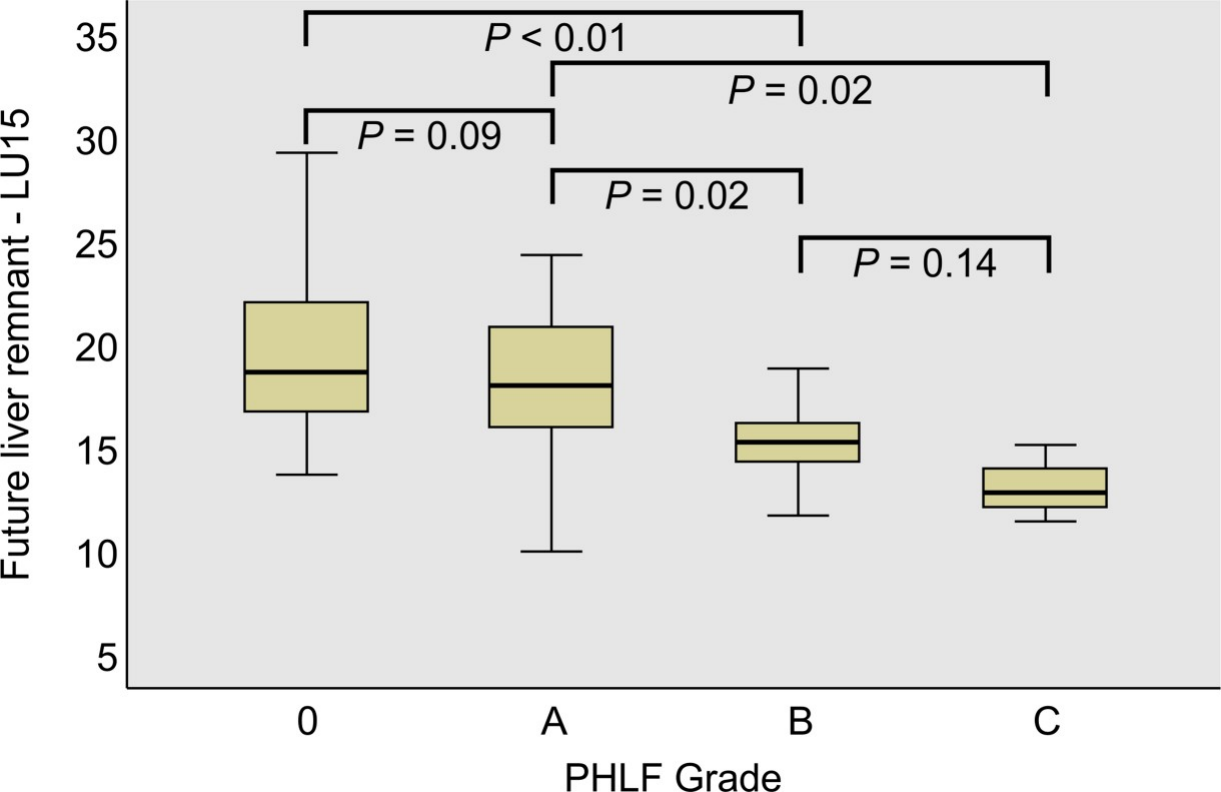
The future liver remnant LU15 values for each posthepatectomy liver failure (PHLF) grade. The differences between stages were evaluated by the Mann–Whitney U test.

The relationship between the FLR-LU15 index and the PHLF grades was evaluated (Fig 3). The median FLR-LU15 values were 18.7, 18.0, 15.2, and 12.8 for Grade 0, A, B, and C, respectively, resulting in a statistically significant difference according to the Kruskal–Wallis test (*P* = 0.01). The differences between each grade were significant for Grades A and B (*P* = 0.02), Grades 0 and C (*P* < 0.01), and Grades A and C (*P* = 0.02) as determined by the Mann–Whitney U test.

### Performance of the FLR-LU15 index as a predictor of PHLF

**Fig 4:**
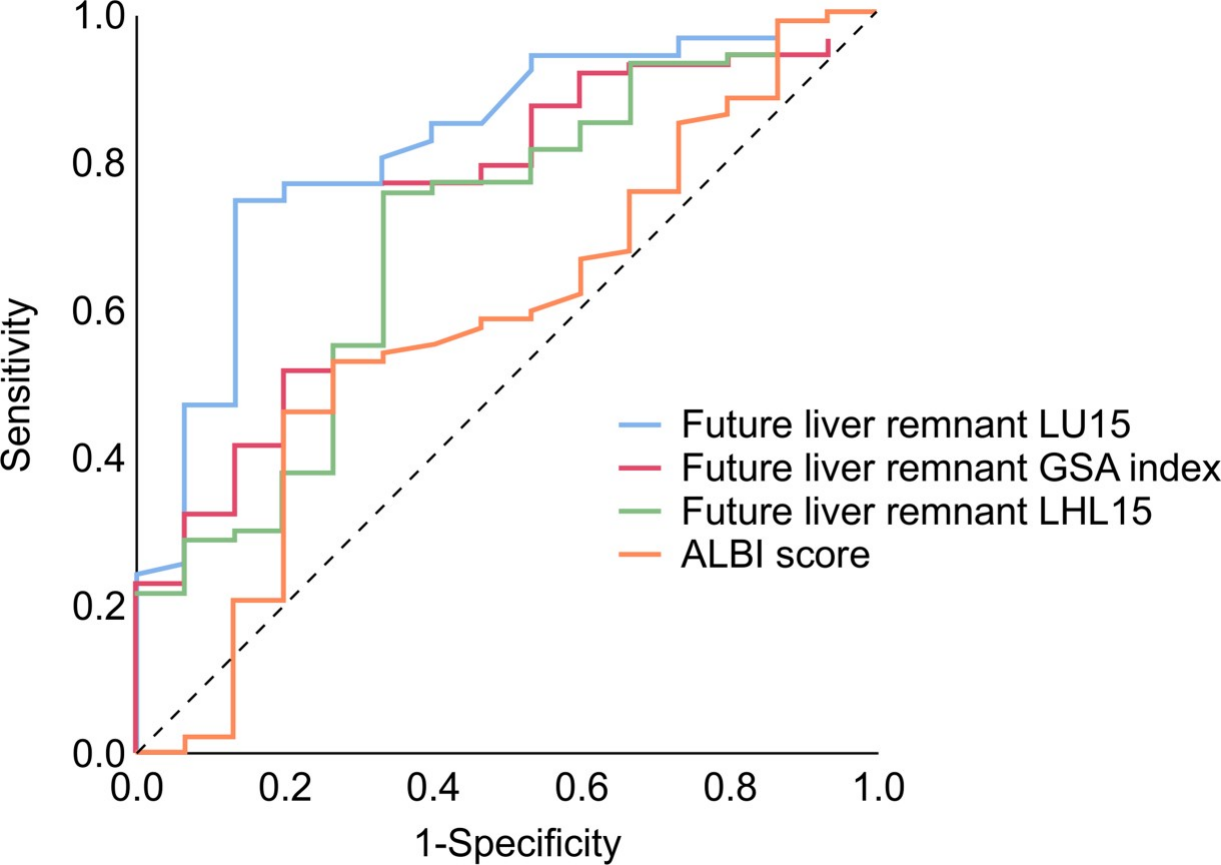
Receiver operating characteristic (ROC) analysis of each ^99m^Tc-galactosyl serum albumin (GSA) scintigraphy value and ALBI (albumin-bilirubin) score for predicting posthepatectomy liver failure (PHLF) ≥ Grade B.

The ROC curves for the FLR-LU15, LHL15, GSA indices and for the ALBI score were compared (Fig 4). The AUROC values of each index are displayed in Table 3. The AUROC for the FLR-LU15 was 0.816, the GSA index was 0.725, the LHL15 was 0.696, and the ALBI score was 0.579. Of these indices, the LU15 had the highest AUROC, which was superior to that of other indices in predicting PHLF.

**Table 3:** Receiver operating characteristic analysis of preoperative factors in predicting the PHLF ≥ Grade B.

	AUROC	95% CI
Future liver remnant LU15	0.816	0.704–0.929
Future liver remnant GSA index	0.725	0.590–0.860
Future liver remnant LHL15	0.696	0.551–0.841
ALBI score	0.579	0.415–0.743

ALBI: Albumin-bilirubin; AUROC: Area under the receiver operation characteristic curve; CI: Confidence interval; GSA index: LHL15/HH15; LHL15: Ratio of uptake by the liver to that by the liver and heart combined at 15 min; LU15: Ratio of uptake by the liver at 15 min to the injected dose of ^99^mTc-GSA; PHLF: Posthepatectomy liver failure.

### Cut-off value of FLR-LU15 index according to PPV and NPV

**Fig 5:**
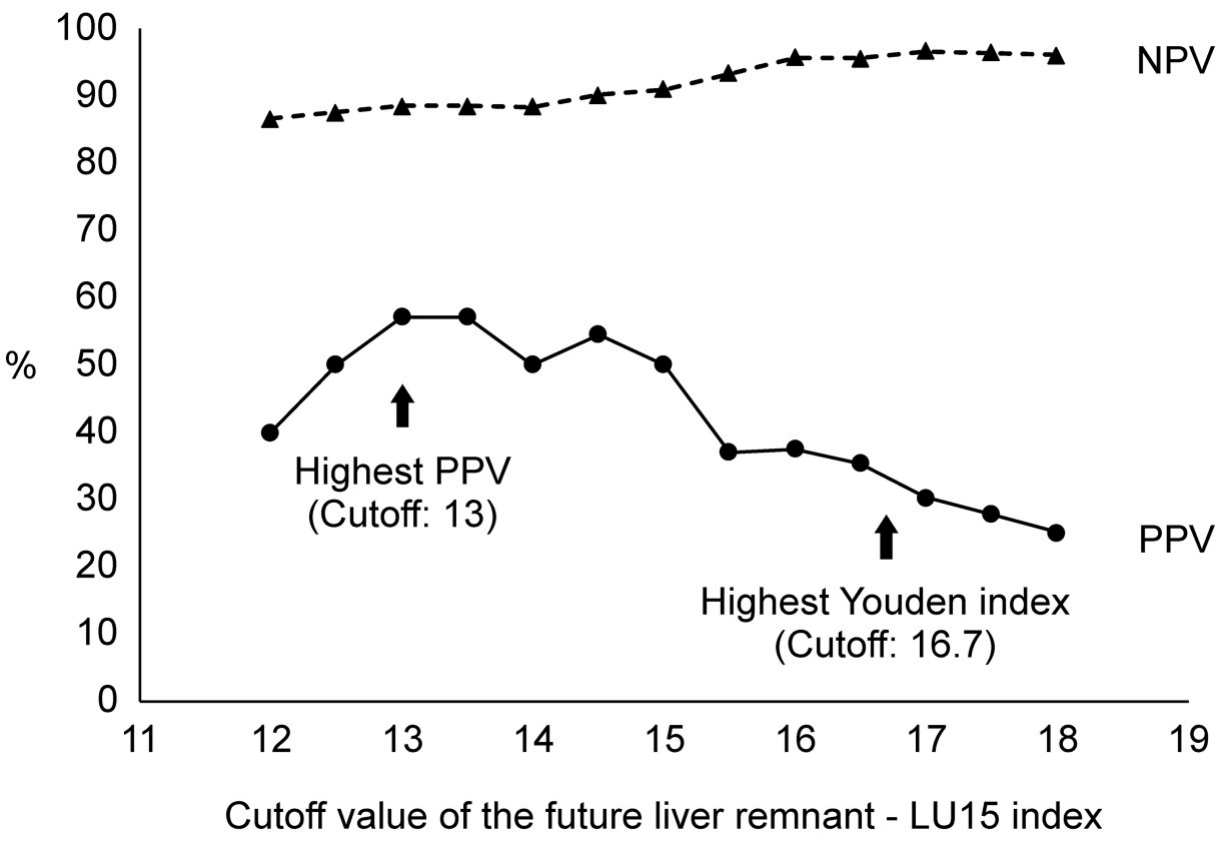
Positive predictive value (PPV) and negative predictive value (NPV) for PHLF ≥ Grade B at each cut-off level of the future liver remnant LU15 index.

The cut-off values of FLR-LU15 for assessing PHLF were evaluated. The effects of changing PPV and NPV according to the cut-off values of the FLR-LU15 index are shown in Fig 5. The cut-off value of 16.7 provided the highest Youden index of the ROC analysis, with a PPV of 37.1% and an NPV of 97%. The cut-off value of 13 provided the highest PPV (57.1%).

### Performance of preoperative parameters as predictors of PHLF

The cut-off values, sensitivities, specificities, PPV, NPV, accuracy, OR, and *P*-values of the FLR-LU15 index, LHL15, GSA index, and the Makuuchi criteria for assessing PHLF are displayed in Table 4. The power of the FLR-LU15 for predicting PHLF was superior to that of the other predictors. When the cut-off value was 16.7, the FLR-LU15 index showed an increased OR of 19.2 (95% CI 4.0–90.9) and an increased NPV of 97.0%, which was useful to exclude the diagnosis of PHLF ≥ Grade B. When the cut-off value was 13, the FLR-LU15 index showed the highest PPV at 57.1%, which represented the lower limit of the FLR-LU15 index for the prevention of PHLF ≥ Grade B.

**Table 4:** Values for the diagnosis of PHLF.

	Cut-off Value	Sensitivity (%)	Specificity (%)	PPV (%)	NPV (%)	Accuracy (%)	Odds Ratio	95% CI	*P–*value
Future liver remnant LU15	16.7	86.7	74.7	37.1	97.0	76.4	19.2	4.0–90.9	< 0.01
Future liver remnant LU15	13	26.7	96.6	57.1	88.4	86.2	10.2	2.0–52.6	0.01
Future liver remnant LHL15	0.58	66.7	75.9	32.3	93.0	74.5	6.3	1.9–20.4	< 0.01
Future liver remnant GSA index	0.88	66.7	77.0	33.3	93.1	75.5	6.7	2.0–21.7	< 0.01
Makuuchi criteria	–	40.0	80.5	26.1	88.6	74.5	2.7	0.9–8.8	0.08

CI: Confidence interval; GSA index: LHL15/HH15; LHL15: Ratio of uptake by the liver to that by the liver and heart combined at 15 min; LU15: Ratio of uptake by the liver at 15 min to the injected dose of ^99^mTc-GSA; NPV, Negative predictive value; PHLF: Posthepatectomy liver failure; PPV: Positive predictive value.

## Discussion

PHLF is a serious complication of liver resection. To prevent PHLF, an accurate assessment of preoperative liver function is essential, and identification of specific indicators may contribute to the prediction of PHLF.

There have been several studies and reports describing methods for the preoperative prediction of PHLF, including the ICG test with ICG-R15 and KICG [16], Child-Pugh score, liver damage classification, Makuuchi criteria [17], ALBI grades [18], ALICE grades [19], measurement of serum hyaluronic acid levels, maximum liver function capacity test [20], and asialoglycoprotein receptor analysis [21]. Araki et al. [22] reported that Gd-EOB-DTPA-enhanced magnetic resonance imaging was useful for predicting PHLF. Other reports mentioned the usefulness of ^99m^Tc-mebrofenin scintigraphy for predicting PHLF [23, 24]. Even though these methods appear to be useful for the estimation of liver function, the precise prediction and prevention of PHLF remain difficult, and a more accurate method for predicting PHLF is still needed.

The efficacy of ^99m^Tc-GSA scintigraphy in assessing liver function has been reported. In ^99m^Tc-GSA scintigraphy, GSA binds specifically to asialoglycoprotein receptors [25, 26], reflecting the number and function of hepatocytes [11, 27]. One of the strong benefits of ^99m^Tc-GSA scintigraphy is the possibility of calculating FLR function before surgery. This is useful in patients who have undergone PTPE or repeat hepatectomy because their hepatic function is not uniform across different hepatic regions. Most of the other indices are difficult to use for calculating FLR function. The KICG can estimate the value of the FLR [28], however, it is calculated by simply multiplying the total liver function by future liver volume, and FLR cannot be calculated directly. Whereas, ^99m^Tc-GSA scintigraphy can measure the FLR directly by fusion of 3D-simulated images, built from enhanced dynamic CT scans, and ^99m^Tc-GSA scintigraphy. ^99m^Tc-mebrofenin scintigraphy can also calculate the FLR directly [24], however, the mebrofenin is excreted in the bile after 10–15 min of administration, and it is difficult to perform dynamic scintigraphy. Additionally, ^99m^Tc-GSA scintigraphy can be performed in both normal liver and cirrhosis in the same way, and the same cut-off value can be applied for HCC and liver metastasis. Furthermore, ^99m^Tc-GSA scintigraphy can be performed in patients with jaundice or hyperbilirubinemia because the asialoglycoprotein receptor is not affected by serum bilirubin levels. This is one of the advantages of ^99m^Tc-GSA scintigraphy compared to other indices, such as ICG test and ^99m^Tc-mebrofenin scintigraphy.

^99m^Tc-GSA scintigraphy uses several indices to evaluate the liver function, including the LHL15, HH15, LU15, and GSA. Of these indices, LHL15 and HH15 are the most popular and are widely used in many institutions. However, Koizumi et al. suggested that the LU15 index is a useful indicator [12], and we have previously reported that ^99m^Tc-GSA scintigraphy is especially useful for the prediction and prevention of PHLF [14, 15]. To the best of our knowledge, the present study is the first to assess the usefulness of the FLR-LU15 index in predicting PHLF ≥ Grade B and to report the of cut-off levels. The LU15 value might be superior to other indices of ^99m^Tc-GSA scintigraphy in terms of performance because it is not affected by heart uptake values that may vary according to cardiovascular status or to a specific region of interest [29, 30]. Furthermore, it has a strong correlation with ICG-R15 values [12].

This study has several limitations, and a potential for bias. First, it is a retrospective study. A multicenter prospective study is warranted to validate the efficacy of ^99m^Tc-GSA scintigraphy, including FLR-LU15, in predicting PHLF. Second, there is a potential impact of selection bias. The results of the study cannot be directly extrapolated and generalized to other populations, including patients with partial hepatectomy or those with biliary or vascular reconstruction, together with hepatopancreatoduodenectomy, in whom the incidence of PHLF has a slightly weaker correlation with the result of ^99m^Tc-GSA scintigraphy; in such conditions other predictive systems would have to be investigated. Third, this study could not predict PHLF Grade C because of the small number of patients with severe PHLF. Therefore, additional studies investigating ^99m^Tc-GSA scintigraphy are needed to achieve a better prediction of PHLF.

## Conclusion

The LU15 value in ^99m^Tc-GSA scintigraphy is a useful indicator of PHLF. The FLR-LU15 should be ≥ 13, and should not be < 16.7 for predicting PHLF ≥ Grade B. This promising method of predicting PHLF in hepatectomy patients may contribute to improvements in posthepatectomy outcomes.

## References

[1] Schroeder RA, Marroquin CE, Bute BP, Khuri S, Henderson WG, Kuo PC. Predictive indices of morbidity and mortality after liver resection. Ann Surg. 2006;243: 373–379. 10.1097/01.sla.0000201483.95911.08 16495703 PMC1448949

[2] van den Broek MA, Olde Damink SW, Dejong CH, Lang H, Malagó M, Jalan R, et al Liver failure after partial hepatic resection: definition, pathophysiology, risk factors and treatment. Liver Int. 2008;28: 767–780. 10.1111/j.1478-3231.2008.01777.x 18647141

[3] Schindl MJ, Redhead DN, Fearon KC, Garden OJ, Wigmore SJ; Edinburgh Liver Surgery and Transplantation Experimental Research Group (eLISTER). The value of residual liver volume as a predictor of hepatic dysfunction and infection after major liver resection. Gut. 2005;54: 289–296. 10.1136/gut.2004.046524 15647196 PMC1774834

[4] Kubota K, Aoki T, Kumamaru H, Shiraki T, Miyata H, Seto Y, et al Use of the National Clinical Database to evaluate the association between preoperative liver function and postoperative complications among patients undergoing hepatectomy. J Hepatobiliary Pancreat Sci. 2019;26: 331–340. 10.1002/jhbp.644 31211911

[5] Eguchi H, Umeshita K, Sakon M, Nagano H, Ito Y, Kishimoto SI, et al Presence of active hepatitis associated with liver cirrhosis is a risk factor for mortality caused by posthepatectomy liver failure. Dig Dis Sci. 2000;45: 1383–1388. 10.1023/a:1005564205755 10961718

[6] Narita M, Oussoultzoglou E, Bachellier P, Jaeck D, Uemoto S. Post-hepatectomy liver failure in patients with colorectal liver metastases. Surg Today. 2015;45: 1218–1226. 10.1007/s00595-015-1113-7 25628126

[7] Mao Y, Du S, Ba J, Li F, Yang H, Lu X, et al Using dynamic 99mTc-GSA SPECT/CT fusion images for hepatectomy planning and postoperative liver failure prediction. Ann Surg Oncol. 2015;22: 1301–1307. 10.1245/s10434-014-4117-4 25294018

[8] Kaibori M, Ha-Kawa SK, Maehara M, Ishizaki M, Matsui K, Sawada S, et al Usefulness of Tc-99m-GSA scintigraphy for liver surgery. Ann Nucl Med. 2011;25: 593–602. 10.1007/s12149-011-0520-0 21800021

[9] Kubo S, Shiomi S, Tanaka H, Shuto T, Takemura S, Mikami S, et al Evaluation of the effect of portal vein embolization on liver function by 99mTc-galactosyl human serum albumin scintigraphy. J Surg Res. 2002;107: 113–118. 10.1006/jsre.2002.6503 12384072

[10] Beppu T, Hayashi H, Okabe H, Masuda T, Mima K, Otao R, et al Liver functional volumetry for portal vein embolization using a newly developed 99mTc-galactosyl human serum albumin scintigraphy SPECT-computed tomography fusion system. J Gastroenterol. 2011;46: 938–943. 10.1007/s00535-011-0406-x 21523415

[11] Kwon AH, Ha-Kawa SK, Uetsuji S, Kamiyama Y, Tanaka Y. Use of technetium 99m diethylenetriamine-pentaacetic acid-galactosyl-human serum albumin liver scintigraphy in the evaluation of preoperative and postoperative hepatic functional reserve for hepatectomy. Surgery. 1995;117: 429–434. 10.1016/s0039-6060(05)80063-7 7716725

[12] Koizumi K, Uchiyama G, Arai T, Ainoda T, Yoda Y. A new liver functional study using Tc-99m DTPA-galactosyl human serum albumin: evaluation of the validity of several functional parameters. Ann Nucl Med. 1992;6: 83–87. 10.1007/BF03164647 1320389

[13] Harada K, Mizuguchi T, Katagiri Y, Kawamoto M, Nakamura Y, Meguro M, et al Area between the hepatic and heart curves of (99m)Tc-galactosyl-human serum albumin scintigraphy represents liver function and disease progression for preoperative evaluation in hepatocellular carcinoma patients. J Hepatobiliary Pancreat Sci. 2012;19: 667–673. 10.1007/s00534-011-0486-2 22179579

[14] Chiba N, Shimazu M, Takano K, Oshima G, Tomita K, Sano T, et al Predicting hepatic failure with a new diagnostic technique by preoperative liver scintigraphy and computed tomography: a pilot study in 123 patients undergoing liver resection. Patient Saf Surg. 2017;11: 29 10.1186/s13037-017-0143-z 29270223 PMC5735932

[15] Chiba N, Yokozuka K, Ochiai S, Gunji T, Okihara M, Sano T, et al The diagnostic value of 99m-Tc GSA scintigraphy for liver function and remnant liver volume in hepatic surgery: a retrospective observational cohort study in 27 patients. Patient Saf Surg. 2018;12: 15 10.1186/s13037-018-0161-5 29881460 PMC5985586

[16] Rahbari NN, Garden OJ, Padbury R, Brooke-Smith M, Crawford M, Adam R, et al Posthepatectomy liver failure: a definition and grading by the International Study Group of Liver Surgery (ISGLS). Surgery. 2011;149: 713–724. 10.1016/j.surg.2010.10.001 21236455

[17] Makuuchi M, Kosuge T, Takayama T, Yamazaki S, Kakazu T, Miyagawa S, et al Surgery for small liver cancers. Semin Surg Oncol. 1993;9: 298–304. 10.1002/ssu.2980090404 8210909

[18] Johnson PJ, Berhane S, Kagebayashi C, Satomura S, Teng M, Reeves HL, et al Assessment of liver function in patients with hepatocellular carcinoma: a new evidence-based approach-the ALBI grade. J Clin Oncol. 2015;33: 550–558. 10.1200/JCO.2014.57.9151 25512453 PMC4322258

[19] Kokudo T, Hasegawa K, Amikura K, Uldry E, Shirata C, Yamaguchi T, et al Assessment of preoperative liver function in patients with hepatocellular carcinoma—The Albumin-Indocyanine Green Evaluation (ALICE) Grade. PLoS One. 2016;11: e0159530 10.1371/journal.pone.0159530 27434062 PMC4951137

[20] Stockmann M, Lock JF, Riecke B, Heyne K, Martus P, Fricke M, et al Prediction of postoperative outcome after hepatectomy with a new bedside test for maximal liver function capacity. Ann Surg. 2009;250: 119–125. 10.1097/SLA.0b013e3181ad85b5 19561474

[21] Kokudo N, Vera DR, Tada K, Koizumi M, Seki M, Matsubara T, et al Predictors of successful hepatic resection: prognostic usefulness of hepatic asialoglycoprotein receptor analysis. World J Surg. 2002;26: 1342–1347. 10.1007/s00268-002-6262-3 12297928

[22] Araki K, Harimoto N, Kubo N, Watanabe A, Igarashi T, Tsukagoshi M, et al Functional remnant liver volumetry using Gd-EOB-DTPA-enhanced magnetic resonance imaging (MRI) predicts post-hepatectomy liver failure in resection of more than one segment. HPB (Oxford). 2020;22: 318–327. 10.1016/j.hpb.2019.08.002 31477460

[23] Dinant S, de Graaf W, Verwer BJ, Bennink RJ, van Lienden KP, Gouma DJ, et al Risk assessment of posthepatectomy liver failure using hepatobiliary scintigraphy and CT volumetry. J Nucl Med. 2007;48: 685–692. 10.2967/jnumed.106.038430 17475954

[24] Chapelle T, Op De Beeck B, Huyghe I, Francque S, Driessen A, Roeyen G, et al Future remnant liver function estimated by combining liver volumetry on magnetic resonance imaging with total liver function on (99m)Tc-mebrofenin hepatobiliary scintigraphy: can this tool predict post-hepatectomy liver failure? HPB (Oxford). 2016;18: 494–503. 10.1016/j.hpb.2015.08.002 27317953 PMC4913132

[25] Sawamura T, Nakada H, Hazama H, Shiozaki Y, Sameshima Y, Tashiro Y. Hyperasialoglycoproteinemia in patients with chronic liver diseases and/or liver cell carcinoma. Asialoglycoprotein receptor in cirrhosis and liver cell carcinoma. Gastroenterology. 1984;87: 1217–1221. 6092193

[26] Galli G, Maini CL, Orlando P, Deleide G, Valle G. A radiopharmaceutical for the study of the liver: 99mTc-DTPA-asialo-orosomucoid. I: radiochemical and animal distribution studies. J Nucl Med Allied Sci. 1988;32: 110–116. 3049961

[27] Kwon AH, Ha-Kawa SK, Uetsuji S, Inoue T, Matsui Y, Kamiyama Y. Preoperative determination of the surgical procedure for hepatectomy using technetium-99m-galactosyl human serum albumin (99mTc-GSA) liver scintigraphy. Hepatology. 1997;25: 426–429. 10.1002/hep.510250228 9021958

[28] Nagino M, Kamiya J, Nishio H, Ebata T, Arai T, Nimura Y. Two hundred forty consecutive portal vein embolizations before extended hepatectomy for biliary cancer: surgical outcome and long-term follow-up. Ann Surg. 2006;243: 364–372. 10.1097/01.sla.0000201482.11876.14 16495702 PMC1448943

[29] Ogasawara G, Inoue Y, Itoh Y, Tagami S, Matsunaga K, Miki K. Improved reproducibility of simple quantitative indices from ⁹⁹mTc-GSA liver functional imaging. Ann Nucl Med. 2013;27: 487–491. 10.1007/s12149-013-0689-5 23595899 PMC3672508

[30] Koizumi M, Yamada Y, Nomura E, Takiguchi T, Kokudo N. An easy and reproducible semi-automatic method for the evaluation of 99mTc-galactosyl human serum albumin. Ann Nucl Med. 1997;11: 345–348. 10.1007/BF03165305 9460529

